# Flagellar-based motility accelerates IgA-mediated agglutination of *Salmonella* Typhimurium at high bacterial cell densities

**DOI:** 10.3389/fimmu.2023.1193855

**Published:** 2023-05-18

**Authors:** Samantha K. Lindberg, Graham G. Willsey, Nicholas J. Mantis

**Affiliations:** ^1^ Department of Biomedical Sciences, University at Albany School of Public Health, Albany, NY, United States; ^2^ Division of Infectious Diseases, Wadsworth Center, New York State Department of Health, Albany, NY, United States

**Keywords:** antibody, IgA, *Salmonella*, agglutination, motility, cyclic-di-GMP, enteric

## Abstract

**Introduction:**

Secretory IgA (SIgA) protects the intestinal epithelium from enteric pathogens such as Salmonella enterica serovar Typhimurium (STm) through a process known as immune exclusion, where invading bacteria are aggregated via antibody cross-linking, encased in mucus, and then cleared from the intestinal tract via peristalsis. At high cell densities, the STm aggregates form a tightly packed network that is reminiscent of early bacterial biofilms. However, the underlying mechanism of how SIgA mediates this transition from a motile and invasive state to an avirulent sessile state in STm is currently unknown.

**Methods:**

In this report, we developed and validated a methodology known as the “snow globe” assay to enable real-time imaging and quantification of STm agglutination by the mouse monoclonal IgA Sal4.

**Results:**

We observed that agglutination in the snow globe assay was dose-dependent, antigen-specific, and influenced by antibody isotype. We determined that flagellar-based motility was a prerequisite for rapid onset of agglutination, even at high cell densities where cell-cell contacts are expected to be frequent. We also investigated the roles of individual cyclic-di-GMP metabolizing enzymes previously implicated in motility and biofilm formation in Sal4 IgA-mediated agglutination.

**Discussion:**

Taken together, our results demonstrate that IgA-mediated agglutination is a dynamic process influenced by bacterial motility and cell-cell collisions. We conclude that the snow globe assay is a viable platform to further decipher the molecular and genetic determinants that drive this interaction.

## 1 Introduction

Immunoglobulin A (IgA) is the predominant antibody isotype in gastrointestinal secretions, where it plays a central role in intestinal homeostasis and mucosal immunity against enteric pathogens (1–3). In the case of the foodborne pathogen *Salmonella enterica* serotype Typhimurium (STm), IgA antibodies against the O-antigen (O-Ag) component of lipopolysaccharide (LPS) molecules expressed on the bacterial surface inhibit adherence to and invasion of intestinal epithelial cells (IECs) (4–6). The molecular mechanisms by which IgA entraps STm in the intestinal lumen are not fully understood but likely involve a form of antibody-mediated clumping, or agglutination, of bacterial cells known as immune exclusion (7, 8). Moor and colleagues described a phenomenon they refer to as “enchained growth” in which IgA crosslinks STm daughter cells and prevents their separation after division (4). This form of clumping only afflicts actively dividing cells and is predominant at low (<10^7^ CFU/g) cell densities, which reflects the bacterial burden in a typical enteric infection (9). Another form of agglutination involves the crosslinking of adjacent bacteria through the formation of antibody-mediated intercellular bridges. This type of aggregation, referred to as “classical agglutination”, occurs only at sufficiently high cell densities where cell-cell contacts can occur. Once initiated, however, classical agglutination of STm results in the formation of macroscopic bacterial mats that share many of the hallmarks associated with bacterial biofilms (6, 10–13). Understanding how IgA drives the conversion of STm from a highly invasive pathogenic modality to an aggregated, non-infectious state has implications for mucosal vaccines and therapies aimed at eliminating bacterial reservoirs within the gut.

IgA-mediated agglutination of STm has long been studied in the case of the mouse monoclonal antibody (mAb) Sal4. Sal4 IgA is specific for the STm O5 antigen (O5-Ag) and was derived as a B cell hybridoma from the Peyer’s patches of mice orally vaccinated with attenuated STm (14, 15). Reflecting its mucosal origin, Sal4 IgA is predominately dimeric (15). Indeed, subcutaneous implantation of Sal4 hybridomas into mice resulted in the delivery of Sal4 IgA into the intestinal lumen in the form of SIgA at levels sufficient to protect against STm invasion following intragastric challenge (15). Similar protection is observed when Sal4 IgA and SIgA are administered to mice by gavage, either concurrently with or immediately before STm challenge (5). In a recent study, we demonstrated that dimeric Sal4 IgA is actively transported across human organoid and enteroid-derived IEC monolayers, where it reduced STm infection by >20 fold (16). Taken together, these studies demonstrate that immune exclusion mediated by Sal4 IgA confers protection against STm invasion of epithelial cells.

In this report, we developed and implemented a method that we refer to as the “snow globe” assay to observe and quantify Sal4-mediated agglutination of STm. We demonstrate that agglutination in the snow globe assay is dose-dependent and antigen-specific (for the most part), and that antibody isotype influences aggregate density. Flagellar expression and function were required for rapid onset of Sal4-mediated agglutination, indicating that collisions of motile bacteria drive this process at high cell densities. Finally, Sal4-mediated agglutination occurred independently of individual cyclic-di-GMP metabolizing enzymes previously linked to motility and biofilm formation in the snow globe assay. Our results demonstrate that IgA-mediated agglutination is a dynamic process influenced by both bacterial and host-derived factors, and further studies are necessary to fully characterize the underlying mechanism of Sal4-mediated agglutination of STm.

## 2 Materials and methods

### 2.1 Bacterial strains and growth conditions

*Salmonella enterica* serovar Typhimurium (STm) strains used in this study are shown in **Table 1** and are derived from the type strain 14028s (ATCC, Manassas, VA). Bacterial culturing was performed as described (6, 10). Unless otherwise stated, 5 mL cultures of Luria-Bertani (LB) medium were inoculated with a single isolated colony from a freshly streaked LB agar plate and grown overnight (~16 h) at 37°C with aeration (220 rpm) in a MaxQ 4000 benchtop incubator (ThermoFisher Scientific, Waltham, MA). Overnight cultures were subcultured 1:50 in LB and grown to mid-log phase (OD_600_ of ~0.7) prior to experimentation. Culture optical density at 600 nm (OD_600_) was monitored using a GENESYS 10S UV-Visible spectrophotometer (ThermoFisher Scientific). When appropriate, media was supplemented with kanamycin (50 µg/mL), carbenicillin (100 µg/mL), gentamicin (10 µg/mL), and/or 5-bromo-4-chloro-3-indolyl-beta-D-galacto-pyranoside (X-gal; 40 µg/mL). L-arabinose was added to a final concentration of 0.4% to induce P_BAD_ promoters. Strains carrying temperature-sensitive plasmids were maintained at 30°C (17).

Table 1Bacterial strains and plasmids used in this study.

**Table:** 

Strain	O-Antigen	Genotype	Reference^1^
Derivatives of *Salmonella* Typhimurium 14028s			
GGW377	O5	14028s	ATCC
GGW444	O5	14028s *zjg8101::kan*	(14)
GGW488	O5	Δ*motB::kan*	
SL001	O5	Δ*STM14_2408::kan*	
SL002	O5	Δ*STM14_3275::kan*	
SL003	O5	Δ*STM14_5467::kan*	
SL048	O5	WT + pBAD24-EV	
SL050	O5	WT + pYeaJ	
SL052	O5	WT + pYhjH	
SL094	O5	WT + pKD46	
SL172	O5	WT + pTn7	
SL174	O5	*attTn7::P_A1/04/03_-lacZ-kan*	
SL180	O4	Δ*oafA::kan*	
SL202	O5	Δ*flhC::kan*	
SL204	O5	Δ*fljB::kan* Δ*fliC::gent*	
*Escherichia coli*			
DH5α		*fhuA2* Δ*(argF-lacZ)U169 phoA glnV44 Φ80* Δ*(lacZ)M15 gyrA96 recA1 relA1 endA1 thi-1 hdR17*	NEB

**Table:** 

Plasmid		Description	Reference
pKD13		R6Kγ ori; Kan^R^	(17)
pKD46		Encodes arabinose-inducible λ Red recombinase genes; Carb^R^	(17)
pTn7		Encodes arabinose-inducible Tn7 transposase; Carb^R^	(18)
pUC18-mini-Tn7T-Gm-lacZ		pBR322 ori; Gent^R^	(19)
pGW167		pUC18-R6K-mTn7- P_A1/04/03_-lacZ-Km; Kan^R^	
pBAD24-EV		Arabinose-inducible, pBR322 ori; Carb^R^	(20)
pYeaJ		pBAD24 with *yeaJ* insertion at XbaI and HindIII sites; Carb^R^	(10)
pYhjH		pBAD24 with *yhjH* insertion at XbaI and HindIII sites; Carb^R^	

^1^Strains and plasmids were generated in this study unless otherwise indicated.

### 2.2 Monoclonal antibodies and hybridomas

The Sal4 B cell hybridoma cell line secreting monoclonal polymeric IgA was maintained as described (15, 21). Chimeric Sal4 IgG_1_ was provided by MappBio, Inc (San Diego, CA) and has been previously characterized *in vitro* and *in vivo* (5).

### 2.3 Construction of STm mutant strains

The oligonucleotide primers used to construct plasmids and STm mutant strains are listed in **Table S1**. λ Red recombination was utilized to generate strains with the target gene replaced by an antibiotic resistance cassette as previously described (17). Kanamycin and gentamicin resistance cassettes were amplified from pKD13 (17) or pUC18-mini-Tn7T-Gm-*lacZ* (19), respectively. The resulting PCR products were purified using a DNA Clean & Concentrator-5 kit according to the manufacturer’s instructions (Zymo Research, Irvine, CA). Electrocompetent STm carrying the pKD46 plasmid (SL94) was grown in the presence of 0.4% arabinose (to induce expression of the λ Red recombinase genes encoded by pKD46), washed with 10% glycerol, and transformed with the concentrated PCR product, then recovered in LB for 1 h at 30°C with aeration. Recovered cells were plated onto LB agar containing kanamycin or gentamicin and incubated overnight at 37°C. Recombinants were verified using colony PCR for antibiotic resistance cassette insertion at the correct genomic location (17). The pKD46 plasmid was cured by incubating the recombineered strains for 4 h at 42°C with aeration in the absence of antibiotic selection.

### 2.4 Generation of a *lacZ*-positive strain of STm 14028s

A constitutive *lacZ* cassette was stably integrated into the STm 14028s chromosome via Tn7 transposition, as described (18). To accomplish this, the pUC18-R6k-mtn7-kanR TN7 recombination vector was first modified to carry the *lacZ* ORF regulated by the constitutive *PA1/04/03* promoter. The *lacZ* ORF and *PA1/04/03* promoter were first amplified from pUC18-mtn7-lacZ-gmR and pUC18-R6k-mtn7-ecfp-gmR (19), respectively, using tailed primers listed in **Table S1**. The PCR products were then purified, assembled into pUC18-R6k-mtn7-kanR in-between the *Sac*I and *Xma*I restriction sites with the NEBuilder HiFi DNA Assembly kit, and subsequently transformed into chemically competent *E. coli* DH5α λpiR (NEB, Ipswich, MA). The resulting *lacZ* Tn7-integration plasmid (pGW167) was electrotransformed into a STm 14028s strain harboring pTn7 (SL172) that had been cultured to mid-log-phase in LB supplemented with carbenicillin and 0.4% arabinose (18). Following 2 h recovery at 30°C with aeration, recombinants were then selected on LB agar supplemented with kanamycin (50 µg/mL) and X-gal (40 µg/mL). The Tn7 transposase plasmid (pTn7) was then cured from blue colonies that arose through subsequent culturing in LB at 42°C with aeration in the absence of antibiotic selection.

### 2.5 pYhjH plasmid construction

To generate the *yhjH* overexpressing plasmid pYhjH, the coding sequence of *yhjH* from the STm 14028s genome was PCR amplified with *yhjH-*specific primers containing built-in restriction sites (listed in **Table S1**) and cloned into the *Xba*I and *Hind*III sites of a pBAD24 vector (20). *E. coli* DH5α competent cells were transformed with the resulting *yhjH* construct (NEB). Transformants were selected on LB agar plates containing ampicillin and the sequence of the selected clone was verified by nucleotide sequencing. To construct a WT pYhjH strain, the plasmid was isolated using a QIAprep Spin Miniprep Kit (QIAGEN, Germantown, MD) and transformed into GGW377 by electroporation. Transformants were selected on LB agar containing carbenicillin.

### 2.6 Macroagglutination (“snow globe”) assay

Overnight cultures of STm were sub-cultured 1:50 in fresh media and grown at 37°C with aeration to mid-log phase (OD_600_ of ~0.7). Cells were then collected via centrifugation (6000 x *g*) for 4 min and washed with sterile PBS (pH 7.4). This step was repeated for a total of two wash steps. Cultures were adjusted to an OD_600_ of 1.0 and transferred into a borosilicate glass tube (16 x 25 mm), resulting in ~2.0 x 10^8^ CFU/mL in 5 mL total volume, prior to Sal4 treatment. For mixture experiments, the indicated strains were prepared as described above, combined in a 1:1 ratio, and gently inverted to thoroughly mix prior to antibody treatment. Sal4-mediated agglutination was recorded using an iPhone 6s (Apple, Cupertino, CA) with the ‘TimestampCamera’ application. Timelapse videos were annotated using Canva (Sydney, Australia). To obtain colony forming units (CFUs), 150 µL was taken from the very top of the culture tube at the air/liquid interface, serial diluted five-fold in sterile PBS pH 7.4, and 100 µL from two consecutive dilutions was plated on LB agar and spread using glass plating beads. Plates were incubated at 37°C overnight in a Heratherm IMH60 incubator (ThermoFisher Scientific) overnight and counted the following day using an eCount Colony Counter (Heathrow Scientific, Vernon Hills, IL). CFU counts from the dilution plates were averaged to calculate a CFU/mL value for each tested condition. Plates with >300 CFUs or <30 CFUs (per 100 μL) were considered too numerous or too few to count, respectively, and were excluded from the final data set.

### 2.7 Enzyme-linked immunosorbent assay

Bacterial cultures were prepared as described above and the OD_600_ value of the washed cells was standardized to a value of 1.0 before addition of 100 µL per well to Immulon 4HBX plates (ThermoFisher Scientific). The plate was covered with a plastic lid and incubated at 4°C overnight (~18 h). The next morning, 200 µL of blocking solution (2% goat’s serum in PBS containing Tween-20 [0.1% v/v]) was added to each well and the plate was incubated on a plate rocker (VWR, Radnor, PA) for 2 h at room temperature. The plate was washed three times with PBS-T prior to addition of Sal4 antibodies diluted in blocking solution. The plate was incubated for 1 h at room temperature on a plate rocker and washed again three times. Goat-anti-mouse IgA-HRP secondary antibody (Sigma-Aldrich, St. Louis, MO) was diluted in blocking solution at 1:2000 and 100 µL was added to each well. The plate was incubated for one h at room temperature on a plate rocker and then washed three times prior to development with SureBlue TMB 1-Component Microwell Peroxidase Substrate (SeraCare, Milford, MA). The peroxidase reaction was stopped using 1M phosphoric acid and the absorbance at 450 nm (Abs_450_) was read by a SpectraMax iD3 plate reader (Molecular Devices, San Jose, CA).

### 2.8 Soft agar motility assay

Motility assays were performed essentially as described (21). Liquid LB media with and without antibodies was combined with an equal volume of liquified 0.6% LB agar, poured into a 100 x 15 mm square grid petri dish (ThermoFisher Scientific), and allowed to solidify at room temperature. Agar plates were stab inoculated with 1 µL of an STm overnight culture and then incubated at 37°C until bacterial growth approached the border of a 2x2 square grid. Plates were imaged using a Gel Doc XR Gel Documentation System (Bio-Rad, Hercules, CA) and the bacterial migration diameter was measured using Fiji software version 2.9.0 (22).

### 2.9 Dot blot

STm colonies from freshly streaked agar plates were used to inoculate individual wells of a 96-well microtiter plate each containing 200 µL LB media (CELLTREAT Scientific Products, Pepperell, MA). Plates were incubated for 3 h at 37°C and 220 rpm, then 3 µL from each well was spotted onto a nitrocellulose membrane (Bio-Rad) and allowed to dry for at least 30 minutes in a fume hood at room temperature. The membrane was then submerged in blocking solution and incubated on a plate rocker overnight at 4°C. The membrane was washed 3 times in 0.1% PBS-T for 10 min prior to addition of 10 µg/mL Sal4 diluted in blocking solution. The membrane was incubated for 90 min at room temperature on a plate rocker and washed again three times in PBS-T. The membrane was incubated with goat-anti-mouse IgA-HRP secondary antibody (Sigma-Aldrich) diluted in blocking solution at 1:2000 for 1 h and then washed five times in PBS-T. Finally, SureBlue TMB 1-Component Microwell Peroxidase Substrate (SeraCare, Milford, MA) was applied to the membrane to detect Sal4 binding. The membrane was then imaged using a Gel Doc XR Gel Documentation System (Bio-Rad).

### 2.10 Statistics and graphics

**Figure 1:**
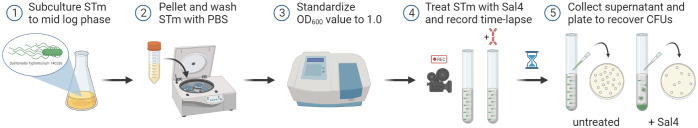
Schematic of the snow globe assay procedure. Cultures of STm are grown to mid-log phase (OD_600_ ~ 0.7), pelleted via centrifugation, washed with PBS, and then the optical density of the cells is standardized to a value of 1.0 prior to treatment with Sal4. Agglutination of the bacterial cells is filmed over time using a timelapse video camera. Finally, the top of the culture supernatant is collected, serial diluted, and plated to quantify the observed agglutination by recovering CFUs.

Statistical analysis was performed using GraphPad Prism 9 software (San Diego, CA). The schematic from **Figure 1** was designed using BioRender.com (Toronto, ON, Canada).

## 3 Results

### 3.1 Real-time visualization and quantification of Sal4 IgA-mediated agglutination of STm

To examine the interaction between STm and Sal4, we developed a method to visualize and quantitate classical IgA-mediated agglutination of STm in real-time, which we refer to as the “snow globe” assay (**Figure 1**). In this assay, STm is grown to mid log phase, harvested, washed, and then resuspended in PBS to reach a final optical density (at 600 nm) of 1.0. Cultures are then transferred to a borosilicate glass tube, treated with Sal4 IgA at 15 µg/mL, and incubated at room temperature for 2 h. Bacterial aggregate formation is then captured with timelapse videography.

**Figure 2:**
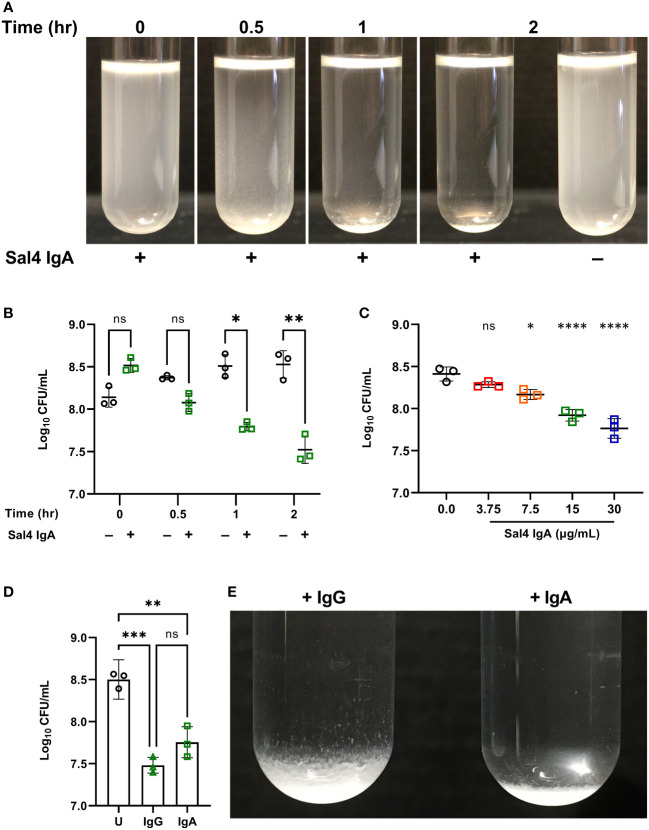
Characterizing Sal4-mediated agglutination of STm 14028s using the snow globe assay. **(A)** Mid-log phase cultures of WT STm were washed in PBS and left untreated or treated with 15 µg/mL of Sal4 IgA for 2 h at room temperature. Representative side-view stills of the WT cultures at the indicated timepoints are shown. **(B)** Mid-log phase cultures of WT STm were washed in PBS and left untreated or treated with 15 µg/mL of Sal4 IgA. Just after antibody addition, and then at 30 minutes, 1 h, and 2 h post-treatment, the top of the supernatant was collected and plated on LB agar to measure CFUs. Data represents base-10 log-transformed CFU/mL values of three biological replicates and error bars represent standard deviation of the mean. Statistical significance was determined by repeated measures two-way ANOVA with Geisser-Greenhouse correction followed by Šidák’s multiple comparisons test. **(C)** WT STm was treated with Sal4 IgA at the indicated concentrations and the top of the supernatant was collected and plated to measure CFUs after 1 h of treatment. Data represents base-10 log-transformed CFU/mL values of three biological replicates and error bars represent the standard deviation of the mean. Statistical significance was determined by one-way ANOVA followed by Dunnett’s *post hoc* multiple comparisons test. Asterisks (*, ****) above treatment groups indicate p < 0.05, p < 0.0001 (respectively) and ns = not significant compared to the untreated control group. **(D)** Mid-log phase cultures of WT STm were washed in PBS and left untreated (circles), treated with 15 µg/mL of Sal4 IgG (triangles), or IgA (squares). After 2 h of treatment, the top of the supernatant was collected and plated on LB agar to measure CFUs. Data represents base-10 log-transformed CFU/mL values of three biological replicates and error bars represent standard deviation of the mean. Statistical significance was determined by one-way ANOVA followed by Tukey’s *post hoc* multiple comparisons test. Asterisks (**, ***) indicate p < 0.01 and p < 0.001, respectively, and ns = not significant. **(E)** Mid-log phase cultures of WT STm were washed in PBS and left untreated or treated with 15 µg/mL of Sal4 IgG or IgA for 2 h at room temperature. Representative side-view stills of both Sal4-treated cultures are shown.

In the absence of Sal4 IgA, wild type (WT) STm remained in suspension for the entire 2 h experimental period (**Figure 2A**; **Movie S1**). The cell suspension was uniformly turbid and free of visible sedimentation at the bottom of the culture tubes. Conversely, the Sal4 IgA-treated cultures appeared to flocculate (“snow”) and aggregate at the bottom of the culture tube within 30 min. Agglutination appeared to occur beginning at the top of the air-liquid interface, with the upper phase (~1-2 cm) of the liquid culture becoming notably more transparent as compared to the untreated control over time. To investigate whether the visible clearing was associated with reduction in colony forming units (CFUs), we removed 150 µL from just below the air-liquid interface of the control and Sal4 IgA-treated cultures and plated on LB agar. In the control cultures, the number of CFU/mL remained relatively constant over the 2 h period, demonstrating that STm does not auto-aggregate or passively settle due to gravity in the absence of Sal4. In contrast, there was a time-dependent reduction in CFUs/mL for the Sal4 IgA-treated cultures that achieved statistical significance within 1 h (**Figure 2B**). At 2 h, there was a ~10-fold reduction in total CFU/mL recovered from the air-liquid interface of cultures treated with Sal4 IgA relative to the control cultures. Nevertheless, ~10^7^ CFUs/mL remained in solution, indicating that either STm cells were not sufficiently coated with Sal4 IgA to promote agglutination of the entire population, or that the cell density dipped (as a result of agglutination) below a threshold required for classical agglutination to effectively occur. Theoretically, classical agglutination is a self-limiting process in situations where aggregates are cleared through settling (as in the snow globe assay) or removed via peristalsis (as would be expected to occur in the gut).

We next titrated Sal4 to determine the effect of antibody concentration on the dynamics of STm agglutination. In the snow globe assay, 7.5, 15, and 30 µg/mL of Sal4 IgA each caused a significant reduction in recovered CFU/mL relative to the untreated control (**Figure 2C**; **Movie S2**). These results demonstrate that STm agglutination by Sal4 IgA is a dose-dependent process in the snow globe assay, consistent with previous reports from our lab (6). To determine the impact of antibody isotype on STm agglutination in the snow globe assay, we utilized a chimeric human IgG_1_ derivative of Sal4 that has been previously evaluated *in vitro* and *in vivo* for effects on STm invasion and agglutination (5, 6). We observed that Sal4 IgG was as effective as Sal4 IgA at reducing CFUs relative in culture supernatants, compared to the untreated control at 2 h (**Figure 2D**). In addition, Sal4 IgG and IgA displayed identical STm flocculation kinetics (**Movie S3**). However, the resulting bacterial sediments were visually distinct; Sal4 IgA-treated aggregates were compact and tightly settled at the bottom of the culture tube, whereas the Sal4 IgG-treated aggregates were less dense and distributed loosely along the walls of the tube (**Figure 2E**). This observation is consistent with previous light microscopic observations that Sal4 IgA induces more densely packed and larger diameter STm aggregates compared to equal concentrations of Sal4 IgG (6). Thus, while Sal4 IgG was as effective as Sal4 IgA at reducing the number STm in suspension, the nature of the aggregates is different between isotypes.

**Figure 3:**
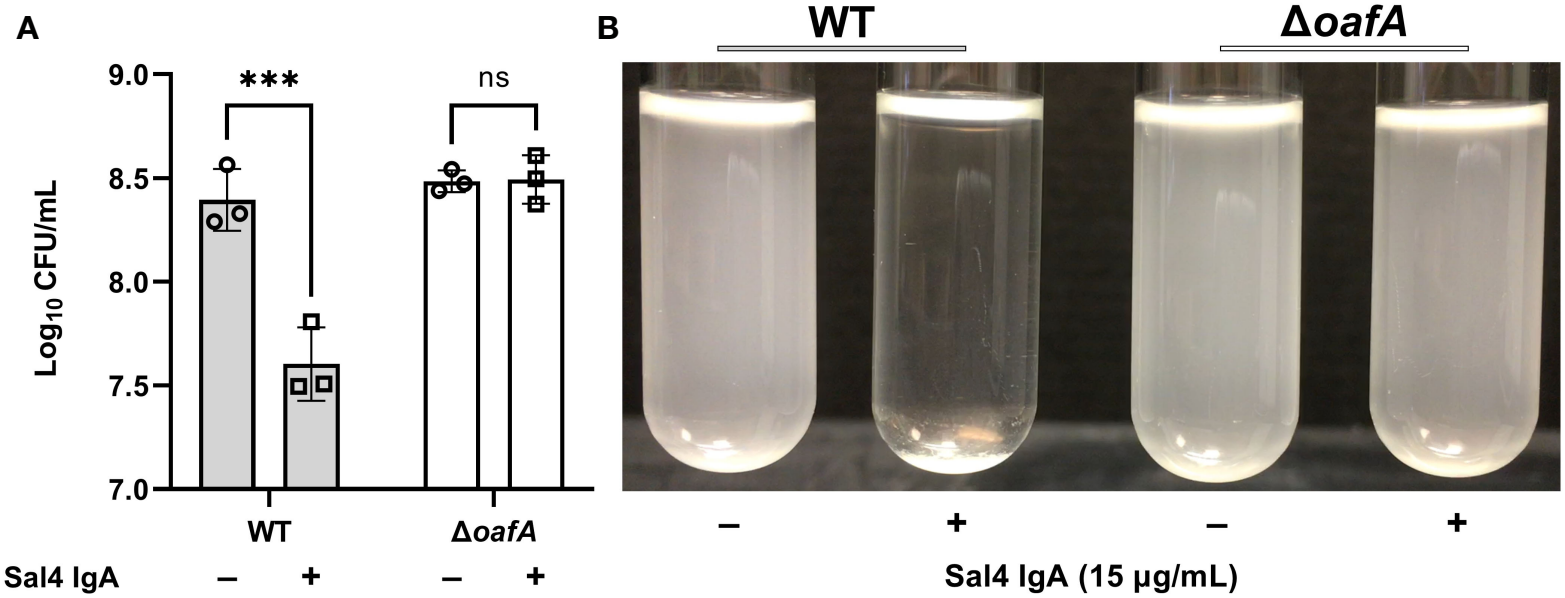
Sal4 IgA agglutinates O5+ STm. **(A)** Mid-log phase cultures of WT (O5^+^) and Δ*oafA* (O5^−^) were washed in PBS and left untreated or treated with 15 µg/mL of Sal4 IgA at room temperature. Representative side-view stills of the cultures at 2 h p.t. are shown. **(B)** Mid-log phase cultures of WT and Δ*oafA* were washed in PBS and left untreated or treated with 15 µg/mL of Sal4 IgA. After 2 h of treatment, the top of the supernatant was collected and plated on LB agar to measure CFUs. Data represents base-10 log-transformed CFU/mL values of three biological replicates and error bars represent standard deviation of the mean. Statistical significance was determined by two-way ANOVA followed by Šidák’s *post hoc* multiple comparisons test. Asterisks (***) indicate p < 0.001 and ns = not significant.

Finally, we confirmed that Sal4 IgA-mediated agglutination of STm in the snow globe assay is dependent on the presence of the O5 antigen. We and others have reported that Sal4 IgA fails to recognize STm strains with deletions in *oafA*, which encodes the O-acetyl transferase that confers the O5 serotype through acetylation of the 2-hydroxyl group of the abequose residue (5, 14, 23, 24). Indeed, we confirmed that Sal4 IgA does not recognize an STm *oafA* mutant via dot blot and ELISA (**Figures S1**, **S3A**). In the snow globe assay, the Δ*oafA* mutant remained in solution and was free of visible flocculation following 2 h of treatment with Sal4 IgA (**Figure 3**; **Movie S4**). Collectively, our results demonstrate that the snow globe assay is a reproducible and quantitative method to monitor STm agglutination in real time.

### 3.2 Flagellar-based motility accelerates Sal4 IgA-mediated agglutination at high cell density

We have previously noted that the process of agglutination of STm by Sal4 IgA is reminiscent of the early stages of biofilm formation (6, 10, 11). Bacterial biofilm development involves a complex interplay between flagellar motility, second messenger c-di-GMP signaling, and cell-cell adhesion (25). To explore these relationships in the snow globe assay, we tested the ability of Sal4 IgA to agglutinate three different motility-deficient STm mutants: Δ*motB*, lacking a component of the flagellar stator complex; Δ*flhC*, absent of the master transcriptional activator of the flagella operons; and Δ*fljB*Δ*fliC*, devoid of both biphasic flagellar filaments (26–29). We confirmed that the three mutants were indeed non-motile in a soft agar motility assay (**Figure S2**). Moreover, all three strains reacted with Sal4 IgA by dot blot analysis, confirming that they express the O5 antigen (**Figure S3A**).

**Figure 4:**
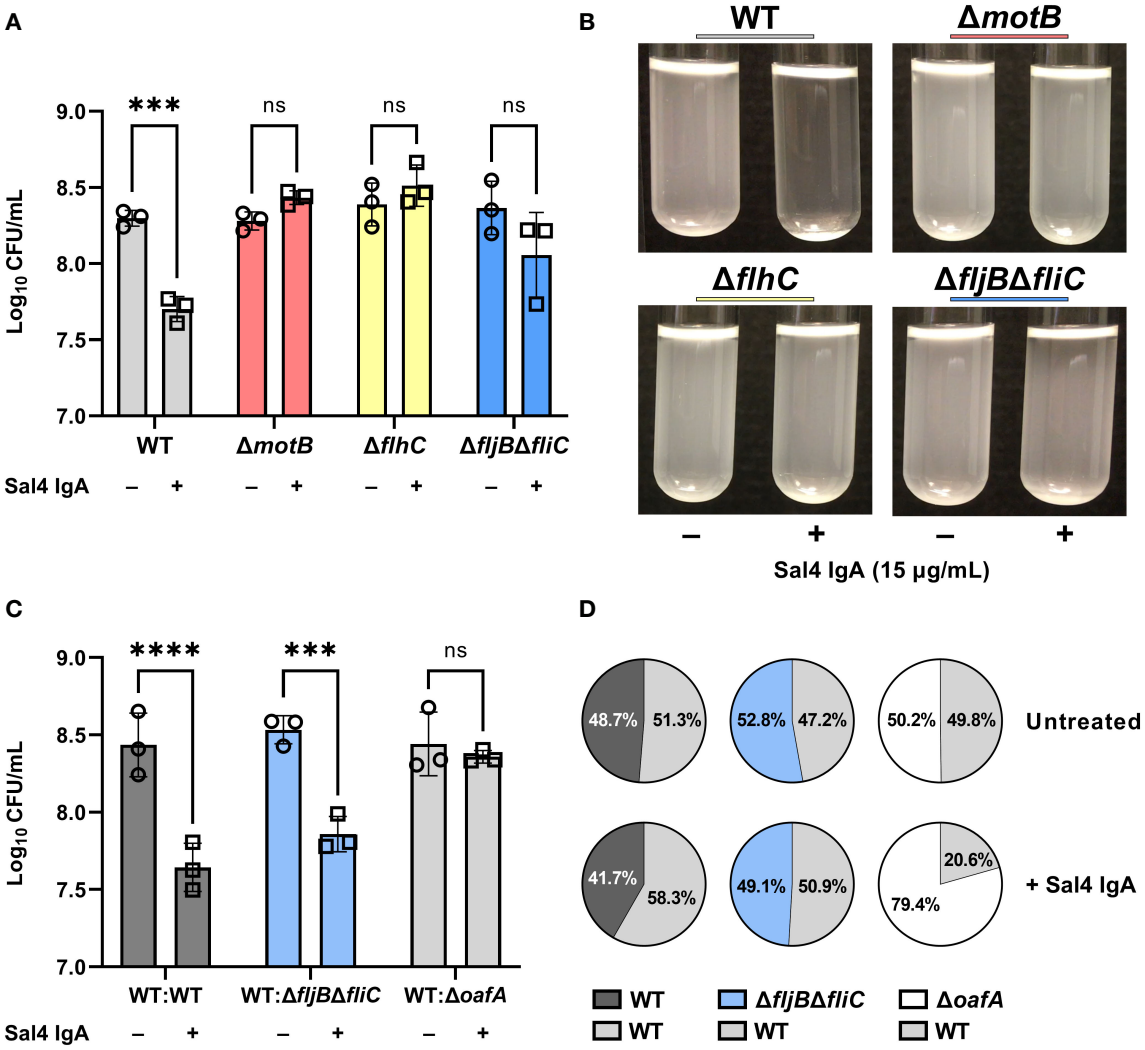
Flagellar-based motility drives Sal4-mediated agglutination of STm. **(A)** Cultures of WT, Δ*motB*, Δ*flhC*, and Δ*fljB*Δ*fliC* were grown to mid-log phase, washed in PBS, and either left untreated or treated with 15 µg/mL of Sal4 IgA. After 2 h of treatment, the top of the supernatant was collected and plated on LB agar to measure CFUs. Representative side-view stills of the cultures at 2 h p.t. are shown. **(B)** Data was obtained from three biological replicates with error bars representing the standard deviation of the mean. Statistical significance was determined by two-way ANOVA followed by Šidák’s *post hoc* multiple comparisons test. Asterisks (***) indicate p < 0.001 and ns = not significant. **(C)** Mixtures of the indicated strains at a 1:1 ratio were either left untreated or treated with 15 µg/mL of Sal4 IgA. After 2 h of treatment, the top of the supernatant was collected and plated on LB agar to measure CFUs. Data was obtained from three biological replicates with error bars representing the standard deviation of the mean. Statistical significance was determined by two-way ANOVA followed by Šidák’s *post hoc* multiple comparisons test. Asterisks (***, ****) indicate p < 0.001 and p < 0.0001, respectively, and ns = not significant. **(D)** Percent composition of the indicated strains present on LB plates with and without Sal4 treatment at 15 μg/mL as determined by blue-white screening. Values represent the average from three biological replicates.

The motility mutants were then evaluated in the snow globe assay in the presence and absence of Sal4 IgA. Over the course of the 2 h experiment, the Δ*motB* and Δ*flhC* mutants remained in solution following Sal4 IgA treatment, with the antibody-treated and untreated cultures indistinguishable from each other (**Figure 4A**; **Movie S5**). The STm Δ*fljB*Δ*fliC* mutant also remained in solution following Sal4 IgA-treatment, although we noted that this culture was somewhat clear at the air-liquid interface and there was a corresponding slight (albeit non-significant) reduction in CFUs recovered relative to the untreated control (**Figure 4B**). None of the untreated cultures displayed any agglutination over the course of 2 h, indicating that these three mutant strains do not passively settle out of solution, regardless of flagellar expression or function (or lack thereof). On the other hand, overnight incubation of these three strains with Sal4 IgA did eventually result in agglutination and pellet formation at the bottom of the culture tube (data not shown), suggesting that the non-motile mutants are not fully resistant to the effects of Sal4 IgA. Rather, we postulate that flagellar-based motility is required for the rapid (<2 h) onset of antibody-mediated agglutination, possibly by increasing the frequency of cell-cell collisions that would nucleate the agglutination process. To test this, we incubated the non-motile Δ*motB* and Δ*fljB*Δ*fliC* mutant strains both statically and with gentle agitation (200 rpm) in the presence and absence of Sal4 IgA for 2 h. We observed that both non-motile mutants moderately agglutinated when combined with Sal4 treatment and agitation, exclusively (**Figure S4**). Taken together, these results indicate that bacterial cell collisions drive antibody-mediated agglutination at high cell density.

### 3.3 Cell-cell collisions and bystander effects in Sal4 IgA-mediated agglutination of STm

We therefore predicted that a non-motile mutant would be prone to agglutination in the presence of a nucleating factor, such as motile WT cells. To test this possibility, we performed a modified snow globe assay in which the non-motile Δ*fljB*Δ*fliC* mutant (*lacZ* ^-^) was mixed 1:1 with an WT strain of STm (*lacZ* ^+^) that constitutively expresses β-galactosidase. The formation of bacterial aggregates was monitored by videography and the proportion of each strain present at the top of the culture was determined by plating CFUs on LB agar containing X-gal. To validate this modified assay, we first compared a *lacZ* ^-^ WT strain mixed with the *lacZ* ^+^ WT strain. The WT : WT mixture behaved as expected, in that visible agglutination occurred following Sal4 IgA treatment and the ratio of the two strains (*lacZ* ^-^; *lacZ* ^+^) in the supernatants after 2 h was roughly 50:50 (**Figures 4C, D**; **Movie S6**). We then examined a 1:1 mixture of STm Δ*fljB*Δ*fliC* (*lacZ* ^-^) with WT STm (*lacZ* ^+^) and observed that the mixed culture agglutinated following Sal4 IgA treatment and there was a statistically significant reduction in CFUs in the culture supernatants relative to the untreated mixed control. Blue-white screening of the resulting colonies indicated that the WT and STm Δ*fljB*Δ*fliC* strains were in equal proportion (**Figures 4C, D**), revealing that a non-motile mutant is effectively agglutinated by Sal4 IgA in the presence of a motile counterpart.

We envisioned two models to explain Sal4 IgA-mediated agglutination of STm Δ*fljB*Δ*fliC* in the presence of WT STm. The first is “bystander catch”, in which WT cells that aggregate following Sal4 IgA exposure entrap non-motile bystander cells in close proximity. We previously reported that STm produces an exopolysaccharide (EPS)-like substance in response to Sal4 IgA treatment, and this could enhance cell-cell adhesion in addition to antibody cross-linking (10, 11, 30). Alternatively, agglutination is driven by the forceful collision of two cells that share Sal4 IgA’s epitope, thereby enabling antibody-mediated intercellular crosslinking or cell-cell bridging to occur, as proposed by Hoces and colleagues (1). To distinguish between these two models, we performed a modified snow globe assay in which the wild type STm (*lacZ* ^+^) was mixed 1:1 with a STm Δ*oafA* (*lacZ* ^-^) mutant. In this experiment, the WT:Δ*oafA* mixture exhibited a partial (or mild) agglutination phenotype and resulted in a markedly less thick/dense sediment at the bottom of the culture tube, as compared to the WT : WT and WT:Δ*fljB*Δ*fliC* mixtures (**Movie S6**). When we examined CFUs, there was no significant reduction in cell numbers from the culture supernatants when compared to the untreated condition, confirming that agglutination of the WT strain was markedly attenuated in the presence of the STm Δ*oafA* (*lacZ* ^-^) strain (**Figure 4C**). This was further supported by the observation that the STm Δ*oafA* (*lacZ* ^-^) mutant outnumbered the WT strain in the cell suspension by ~4 fold with Sal4 treatment (**Figure 4D**). While these results favor the cell collision model noted above, they also suggest that bystander catch does indeed occur at high cell densities.

### 3.4 Sal4-mediated agglutination occurs independently of PDEs/DGCs previously implicated in motility arrest and EPS production

**Figure 5:**
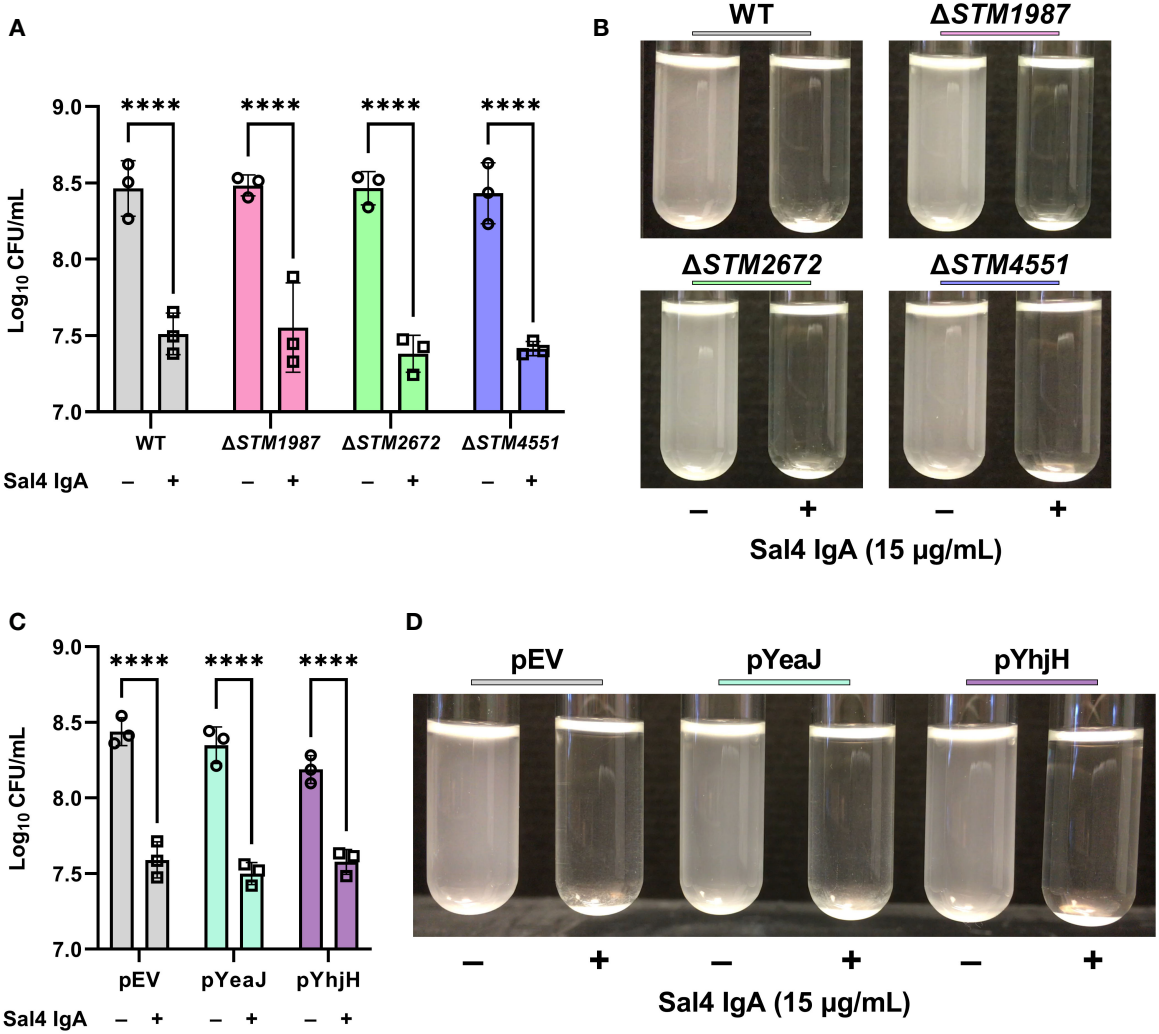
Contribution of c-di-GMP modulating enzymes to Sal4-mediated agglutination. **(A)** Mid-log phase cultures of WT, Δ*STM1987*, Δ*STM2672*, and Δ*STM4551* were washed in PBS and left untreated or treated with 15 µg/mL of Sal4 IgA. After 2 h of treatment, the top of the supernatant was collected and plated on LB agar to measure CFUs. Representative side-view stills of the cultures at 2 h p.t. are shown. **(B)** Quantification of antibody-mediated agglutination. **(C)** WT STm transformed with an arabinose-inducible pBAD24-derived plasmid that overexpresses the diguanylate cyclase gene *yeaJ* (pYeaJ) or the phosphodiesterase gene *yhjH* (pYhjH), or an empty vector control plasmid (pEV), were induced with 0.4% arabinose, grown to mid-log phase, washed in PBS, and either left untreated or treated with 15 µg/mL of Sal4 IgA. After 2 h of treatment, the top of the supernatant was collected and plated on LB agar to measure CFUs. Representative side-view stills of the cultures at 2 h p.t. are shown. **(D)** Quantification of antibody-mediated agglutination. For panels B and D, data was obtained from three biological replicates with error bars representing the standard deviation of the mean. Statistical significance was determined by two-way ANOVA followed by Šidák’s *post hoc* multiple comparisons test. Asterisks (****) indicate p < 0.0001.

We have previously reported that Sal4 IgA triggers STm EPS production and biofilm formation through activation of one or more diguanylate cyclases (DGC) that modulate c-di-GMP signaling in STm (10). Considering the link between c-di-GMP signaling, motility, and biofilm formation, we hypothesized that Sal4 IgA-mediated agglutination in the snow globe assay may be influenced by DGCs previously implicated in motility arrest, including STM14_2408, STM14_3275, and STM14_5467 (referred to hereafter as their LT2 annotations STM1987, STM2672, and STM4551, respectively) (31, 32). To test this hypothesis, we engineered STm strains carrying deletions in *STM1987*, *STM2672*, and *STM4551*, as described in the Materials and Methods, and confirmed reactivity with Sal4 IgA by dot blot (**Figure S3B**). In the snow globe assay, all three mutants were each readily agglutinated by Sal4 IgA (15 μg/mL) with kinetics and changes in CFUs that were virtually identical to that of the WT strain (**Figures 5A, B**; **Movie S7**). Thus, the absence of these DGCs previously implicated in motility arrest does not impact Sal4 IgA-mediated agglutination of STm under the conditions of the snow globe assay.

As the c-di-GMP pool in STm is regulated by a network of multiple DGCs, phosphodiesterases (PDEs), and proteins with both catalytic functions, it is possible that a defect caused by knocking out a single DGC is masked by the activity of the other enzymes. With this in mind, we hypothesized that overexpression of a DGC or PDE might influence Sal4-mediated agglutination of STm. To test this, we utilized previously constructed expression plasmids that encode the DGC *yeaJ* gene or the PDE gene *yhjH* under control of an arabinose-inducible promoter (10). The STm strains carrying these plasmids were confirmed to be reactive with Sal4 IgA via dot blot (**Figure S3C**). In the snow globe assay, the strains overexpressing YeaJ or YhjH were agglutinated by Sal4 in a manner that was indistinguishable from the WT strain carrying the empty vector (**Figures 5C, D**; **Movie S8**). Our results suggest that cyclic-di-GMP signaling does not play a direct role in antibody-mediated agglutination at high cell densities under these experimental conditions.

## 4 Discussion

While the importance of IgA in intestinal immunity to STm and other enteric pathogens is well-established, much remains to be understood about the molecular mechanisms by which luminal antibodies limit bacterial access to epithelial surfaces (4–6, 33). Previous studies have shown that a phenomenon known as enchained growth is principally at play at low cell densities during infection *in vivo* (4). Enchained growth occurs when high-affinity antibodies coat dividing bacteria and prevent cell division, presumably by forming an antibody bridge at the division septum (4). When local cell densities are high, IgA is proposed to function through classical agglutination, in which antibodies crosslink neighboring cells and ultimately cause the formation of rafts that are entrapped in mucus and cleared from the gut via peristalsis (1, 30). In either case, IgA serves as molecular “Velcro” that limits two or more cells from disengaging from each other. Deciphering how IgA promotes and intercellular adhesions and how such interactions are sensed by the bacteria in response is fundamental to understanding host-pathogen interactions in mucosal environments, as well as the onset of biofilm formation.

In this report, we developed and implemented the snow globe assay to visualize and quantify agglutination of STm that occurs following exposure to the anti-LPS monoclonal IgA Sal4 (5, 15, 23). One caveat of this study is that we examined agglutination at a fixed concentration of cells (~10^8^ CFU/mL), which is above the hypothetical infectious dose of STm (4). In addition, the snow globe assay model excludes critical physiological components that STm would encounter in the intestines during infection. Future experiments are necessary to determine the kinetics of Sal4-mediated agglutination at lower cell densities that better reflect the conditions of the gastrointestinal tract. Nevertheless, the snow globe assay proved to be a versatile and sensitive platform in which to begin to dissect underlying genetic factors that influence agglutination dynamics. The snow globe assay was sensitive enough to determine the relative extent of antibody-mediated agglutination between isogenic gene deletion strains of STm 14028s, either individually or in a mixed population, demonstrating the potential application of this method to screen additional mutants. Indeed, the snow globe assay could be implemented to examine the interaction of any given antibody with a variety of STm isolates and surface antigens. For example, Ramachandran et al. isolated polyclonal (hyperimmune sera) and monoclonal antibodies against phase 1 flagellin proteins of *S*. Enteritidis and *S.* Typhimurium that were characterized *in vitro* for bactericidal activity and phagocytic uptake (34). The agglutinative activity of these antibody preparations could be evaluated in the snow globe assay and correlated to *in vivo* protection (35). Furthermore, the snow globe assay can likely be applied to any motile bacterial species, as we have successfully employed it to examine antibody-mediated agglutination of *Vibrio cholerae* in addition to *Salmonella* Typhimurium (data not shown). In contrast to previously implemented methods such as flow cytometry and fluorescence microscopy, the snow globe assay does not require specialized training, which further demonstrates its potential as a simple yet functional assay for a variety of applications (6).

The snow globe assay afforded novel insights into the early events associated with antibody-mediated bacterial agglutination. Sal4 IgA-mediated agglutination of STm was visible within ~20 min and appeared as flocculate (or “snow”) that accumulated as a dense sediment at the base of the borosilicate glass culture tube. The reaction was antigen-specific and influenced by antibody isotype (dIgA versus mIgG). Most notably, we found that non-motile STm mutants were impervious to Sal4 IgA-mediated agglutination, even at high cell densities. Agglutination of a Δ*fljB*Δ*fliC* mutant was “restored” when mixed one-to-one with WT (motile) STm cells, demonstrating that motility serves to nucleate bacterial agglutination. Ultimately, this observation suggests that flagella-based motility is an integral driver of intercellular crosslinking and antibody-mediated agglutination at high cell density. Recent work by Porter and colleagues affords insight into the interplay between motility and the initial stages of agglutination that may be relevant to STm when it encounters Sal4 IgA (36). Specifically, using a model of *E. coli* agglutination driven by high concentrations of the polymer polyethelene glycol (PEG), the authors argue that aggregation comes about when two conditions are met: sufficient inter-bacterial collisions and adequate cell-cell attraction forces. In their model, flagella-based motility drove cell-cell collisions and PEG functioned in cell-cell association via displacement forces.

In the case of STm, we propose that flagella-based motility also drives cell-collisions, while Sal4 IgA functions to tether cells together. This model would explain why the three non-motile STm mutants failed to agglutinate following Sal4 IgA treatment but did so when mixed 1:1 with swimming cells. Within mixed cultures (motile and non-motile cells), it has been proposed that immobile cells serve as nucleation sites, a model that is testable in the case of Sal4 IgA (37). The two-step aggregation model may also account for the observed differences in sediment densities induced by Sal4 IgG and IgA. The Sal4 IgA preparations used in this study are predominantly dimeric (i.e., consisting of two covalently linked IgA monomers), whereas IgG is monomeric. The increased avidity associated with Sal4 IgA as compared to IgG would be expected to have significantly greater attraction forces and therefore result in more densely packed aggregates. Indeed, we have previously observed this phenomenon using *in vitro* derived aggregates (6).

In their review of immune exclusion, Hoces et al. stated that classical agglutination is dependent on high-affinity IgA binding to the bacterial surface and the collisions of “antigenically identical” bacteria (1). Our results are consistent with this model for the most part, as individual and mixed cultures of O5+ strains agglutinated in the presence of Sal4. However, our results suggest that bystander catch can occur as antigenically distinct (O4 versus O5) bacteria were both detected in the pellet of the 1:1 WT:Δ*oafA* mixture when treated with Sal4. Therefore, we cannot exclude the possibility that, at high cell density, WT cells bound to Sal4 entrap and drag down bystander Δ*oafA* cells. We have previously observed that Sal4 treatment results in increased EPS production of WT STm (6, 10, 11). We speculate that ECM production triggered by antibody binding renders cell-cell collisions inescapable, even to immotile or O5^–^ bystanders. Collectively, these results suggest that cellular collisions, and the ECM production caused by Sal4 exposure, supersedes the requirement for antigen presentation, at least to a certain extent (1). Future studies are necessary to determine the contributions of EPS and capsule formation to the flocculation phenotype we observed in this study.

While our studies were limited to examining antibody-mediated agglutination of mixtures of isogenic STm strains in liquid culture, we speculate that the bystander catch phenomenon we observed may occur *in vivo*, especially in environments like the gut where high cell densities exist. Polyclonal and even monoclonal IgA antibodies against surface glycans like LPS have demonstrable cross reactivity within and across bacterial pathogenic and commensal species (38–41). With that in mind, “innocent” bystanders may indeed be entrapped in a bacterial mass consisting of SIgA bound to a pathogenic agent. A motile species may be prone to escape bystander catch, while non-motile bacteria caught in an agglutination cluster may not. However, it is currently unknown if Sal4 IgA has the ability to shape the composition of the microbiome and/or restrict enteric colonization of diverse invading pathogens.

Within 20 minutes of treatment with Sal4, the aggregated cells formed visible flakes and collected at the bottom of the tube, which is reminiscent of flocculation observed in yeast (42). In yeast and bacteria, auto-agglutination is mediated by the expression and activity of cell-surface adhesions (42–44). Based on these studies and our previous observations, we hypothesized that Sal4 triggers agglutination through an analogous intracellular process, such as c-di-GMP signaling (45). Although we did not observe differences in agglutination with individual DGC mutant strains or strains overexpressing c-di-GMP metabolizing enzymes relative to a WT control, the complexity of the c-di-GMP network and the conditions of the snow globe assay could have rendered a potential phenotype imperceptible. The genome of STm encodes at least five DGCs, eight PDEs, and seven proteins that contain both catalytic domains, therefore, deletion of individual CMEs may not be sufficient to alter c-di-GMP signaling in an observable manner (46, 47). Further experiments are necessary to determine the role of c-di-GMP signaling in the collective response to Sal4 exposure.

Although Sal4 effectively reduces invasion *in vivo* and promotes agglutination *in vitro* at 15 µg/mL, we have always been able to detect cells that circumvent these effects. It is possible that there is simply a portion of the culture that coincidentally avoids Sal4 binding and therefore is still motile and able to collide with and adhere to other cells. On the other hand, there may be behavioral mechanisms at play that enable an individual cell to evade Sal4 binding and even resist entrapment by other bacteria. We speculate that individual cells with specific genetic mutations could outcompete other bacteria that are more vulnerable to Sal4. Using the snow globe assay set-up as a platform, we intend to perform a genome-wide screen to identify genetic factors that promote aggregation and cell adhesion in response to Sal4 binding with the goal of further elucidating the mechanism of Sal4-mediated agglutination.

## Data availability statement

The original contributions presented in the study are included in the article/**Supplementary Material**, further inquiries can be directed to the corresponding author.

## Author contributions

Conceptualization: SL, GW, and NM. Methodology: SL and GW. Investigation: SL. Formal analysis: SL. Visualization: SL. Supervision: GW and NM. Funding acquisition: NM. Project administration: NM. Writing – original draft: SL and NM. Writing – review and editing: SL, GW, and NM. All authors contributed to the article and approved the submitted version.
