# Supplementary material for: Flagellar-based motility accelerates IgA-mediated agglutination of Salmonella Typhimurium at high bacterial cell densities
Source: Front Immunol. 2023 May 18;14:1193855. doi: 10.3389/fimmu.2023.1193855 (PMC10232770; doi:10.3389/fimmu.2023.1193855)
Supplement: Supplementary file 9 [file DataSheet_1.pdf]

Supplementary Material

Flagellar-based motility accelerates IgA-mediated agglutination of *Salmonella* Typhimurium at high bacterial cell densities

Samantha K. Lindberg, Graham G. Willsey, Nicholas J. Mantis*

*** Correspondence:** Nicholas J. Mantis: nicholas.mantis@health.ny.gov

1 Supplementary Data

1.1 Supplementary Figures

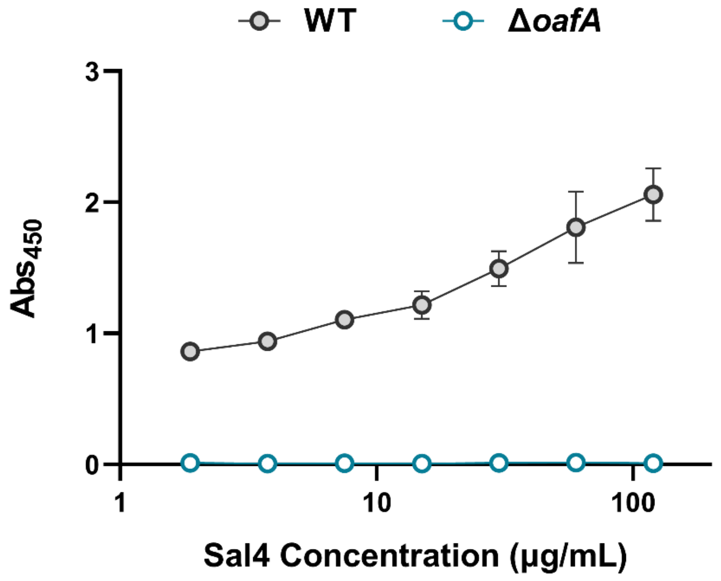

Supplementary Figure 1. Sal4 IgA binds to STm expressing the O5 antigen as measured by ELISA. Mid-log phase cultures of WT (grey circles; $O5^+$) and Δ*oafA* (teal circles; $O5^-$) were washed in PBS, standardized to an OD_{600} of 1.0, and incubated in a 96-well plate at 4°C overnight. Plates were incubated in blocking solution for 2 h, washed with 0.1% PBS-T, and then incubated with Sal4 IgA for 1 h. Plates were washed again, incubated with HRP-conjugated anti-IgA secondary antibodies, and finally developed with TMB substrate, as detailed in the Materials and Methods. Data represents two biological replicates averaged from three technical replicates.

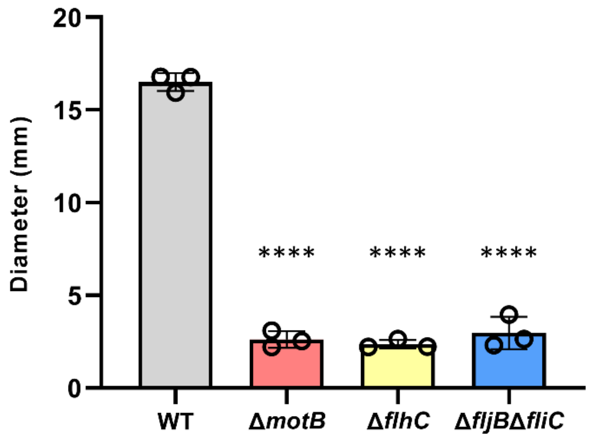

Supplementary Figure 2. Confirmation of reduced swimming in motility-deficient mutant strains. Plates of 0.3% LB agar were stab inoculated with 1.0 µL of overnight cultures of WT, Δ*motB*, Δ*flhC*, and Δ*fljB*Δ*fliC* and then incubated at 37°C for 3.5 h. Plates were then imaged and the diameter (mm) of the bacterial migration was measured using Fiji (version 2.9.0). Data represents three biological experiments each performed with two technical replicates. Statistical significance was determined by ordinary one-way ANOVA followed by Dunnett's post hoc multiple comparisons test. Asterisks (****) indicate $p < 0.0001$ as compared to the WT control group.

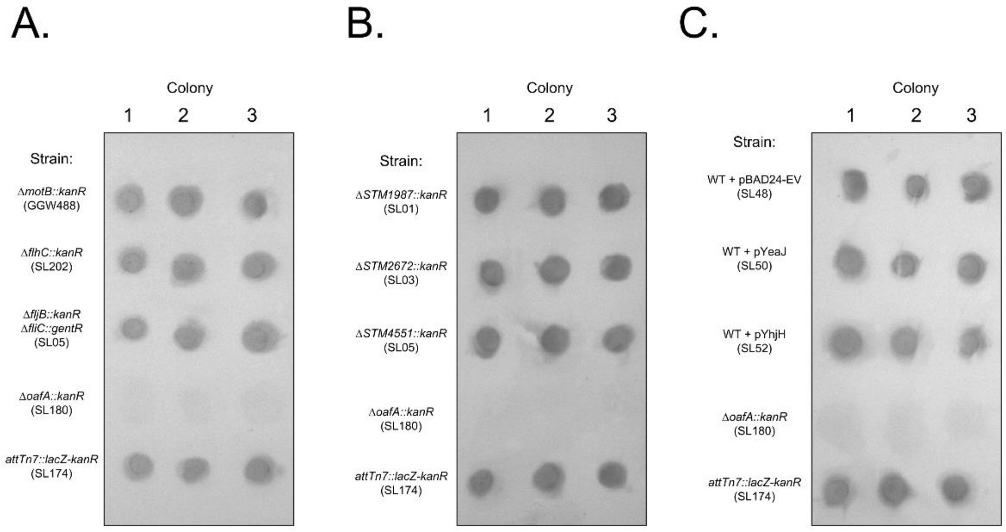

Supplementary Figure 3. Detection of O5 epitope expression for STm strains used in this study. For panels A, B, and C, representative dot blots of the indicated strains are shown. Cultures were grown for 3 hours in a microtiter plate at 37°C with aeration and 3 µL was spotted onto a nitrocellulose membrane. The membrane was allowed to air-dry at room temperature and subsequently incubated in blocking solution at 4°C on a plate rocker overnight. The membrane was washed, incubated with 10 µg/mL Sal4 IgA for 1.5 hours, washed, and then incubated with goat anti-mouse IgA-HRP (alpha-chain specific) secondary antibody for 1 hour. Antibody binding was detected via addition of TMB substrate as detailed in the Materials and Methods. Each dot for each strain represents an individual colony and each assay was performed in duplicate.

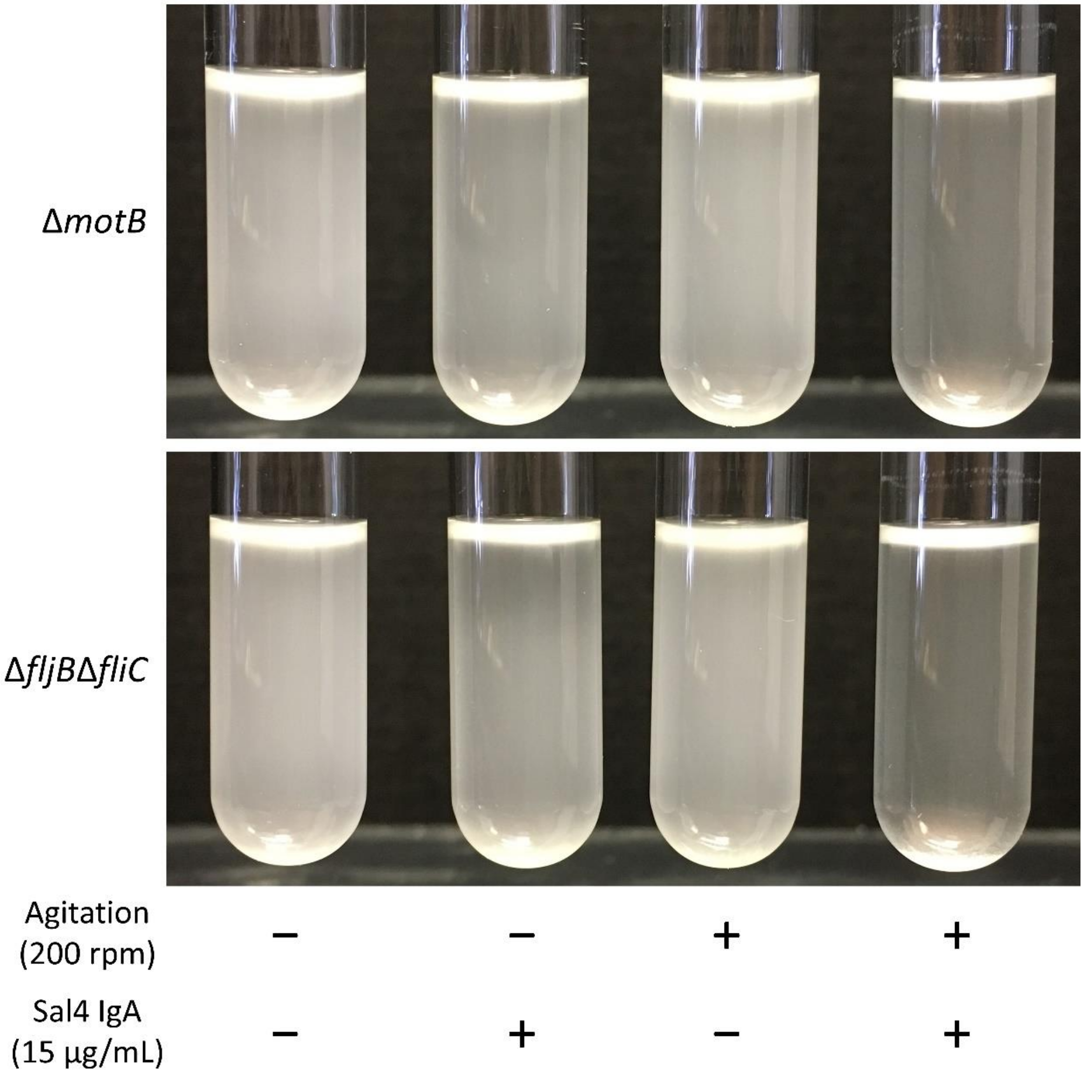

Supplementary Figure 4. Sal4-mediated agglutination of motility-deficient mutants under static and agitated conditions. Cultures of Δ*motB* and Δ*fljB*Δ*fliC* were grown to mid-log phase, washed in PBS, either left untreated or treated with 15 µg/mL of Sal4 IgA, and finally either incubated statically or with gentle agitation (200 rpm) at room temperature for two hours. Representative side-view stills of the cultures at 2 h p.t. are shown and represent two biological replicates.

1.2 Supplementary Table 1. Primers used in this study

Primer Name	Primer Sequence (5' to 3')
flhC_lambda_F	GAA GTG GAC GAT ACG GCG CGT AAG AAA AGG GCA TGA TAT GTG TAG GCT GGA GCT GCT TCG
flhC_lambda_R	ACT TAC CGC TGC TGG AGT GTT TGT CCA CAC CGT TTC GGT AAT TCC GGG GAT CCG TCG ACC
flhC_lambda_screen_F	CAG GTA TCA TGC TTT CAA CGC G
fliC::gentR_lambda_F	CAA TAA CAT CAA GTT GTA ATT GAT AAG GAA AAG ATC CCC CTG ATT CCC TTT GTC AAC AGC
fliC::gentR_lambda_R	TGA TTG TGT ACC ACG TGT CGG TGA ATC AAT CGC CGG AAC TCC GCG GCC GGG AAG CCG ATC
fliC_KO_scrn__F	CTG CCT TCG ACC AAG AAG CGG TTG
fliC_KO_scrnR	CAG GCT CCG GAA TTA AAA AAG GC
fljB_KO_scrn_F	TTA GTC GCT TTT CTC ATG GAG GAT TG
fljB_lambda_F	GGA TTG CTT TAT CAA AAA CCT TCC AAA AGG AAA ATT TTT GTG TAG GCT GGA GCT GCT TCG
fljB_lambda_R	AAG CCC CGA ATT CAC GGG GCT GAA TAA AAC GAA ATA AAT AAT TCC GGG GAT CCG TCG ACC
kanR_R	GAG CGA GCA CGT ACT CGG ATG G
lacZ_gibson_R	GCT TCT CGA GGA ATT CCT GCA GTT ATT TTT GAC ACC AGA CCA ACT GGT AAT GGT AG
lacZorfF	ATG ACC ATG ATT ACG GAT TCA CTG GCC
lambda_Kan_scrnR	GGA TTC ATC GAC TGT GGC CG
motB_lambda_KO_scrn_F	GTA CGT TCC TCG GTA TTT TAC TGG
motB_lambda_SOE_F	ATT GAG TTG GAA GAA CAC GTT CGC GCA GTG AGA AAC CCA AAC CAG CAG CAG ACG ACT GAG GAA GCA TGA GTG TAG GCT GGA GCT GCT TCG
motB_lambda_SOE_R	CAG CCA ACA GCT CGT CAG CTT CAT CAA AAA ATG TCT GAT AAA AAT CGC TAA TAT CCA TGC TCA CGC TAT AAT TCC GGG GAT CCG TCG ACC
oafA_KO_scrn_F	GCG CTG GTC AGT GAC CTT CTT TGA
oafA_lambda_F	ATT TCG TCT TGT GTG GCA CCT TGG AAT TAT AGG TAA AAA GTG TAG GCT GGA GCT GCT TCG
oafA_lambda_R	TGT TGT AGT TTT ATA AAA TAA AAA GAG GGG CAA GCC CCT AAT TCC GGG GAT CCG TCG ACC
PA1/04/03_F_gibson	AAG CTA ATT CGA GAT CAT GCA TGT ACC ATT TAT CAG GGT TAT TGT CTC A
PA1/04/03_R_gibson	GTG AAT CCG TAA TCA TGG TCA TGC TTA ATT TCT CCT CTT TAA TTC TAG ATG
pBAD-F	ATG CCA TAG CAT TTT TAT C
pBAD-R	GAT TTA ATC TGT ATC AGG
STM1987_KO_scrn_F	TAT CGC GCT GAC GCT GTT AT
STM1987_KO_scrn_R	GCG CAA ATA CGG TTA CGT CC
STM1987_lambda_kanR_F	TGA GGT CTG GCT ACC GTA AGC CAT CAG GGG GAG TTG TAT CAA TAA CCA GGA GTT ACC AGG TAA TTC CGG GGA TCC GTC GAC C
STM1987_lambda_kanR_R	CTA TTT CTT TTC CCG CTC CTG AGT CGC GTC GCT GGC GCA AAT ACG GTT ACG TCC TGT GTA GGC TGG AGC TGC TTC G
STM2672_KO_scrn_F	TAT CAC AAC CCG TCT GGC AC
STM2672_KO_scrn_R	AAG CCG TAG CTG CCA TTC TT
STM2672_lambda_kanR_F	TAA TGC TTG CAC GGA ATC AAA AGC ATG AAT AAG GAA TTT TCT CTG TCC AGG CCA ACA TTT TAA TTC CGG GGA TCC GTC GAC C

STM2672_lambda_kanR_R	ATG GTT GCA TGA TTA GGC TGG GTC AGT GGT CTC AAC GCT GAG TCA GAA ACG GCC AGG CCC TGT GTA GGC TGG AGC TGC TTC
STM4551_KO_scrn_F	TCT TTG GGG GAA TAT GGG CG
STM4551_KO_scrn_R	TCA GGC CAT TGA GGG TTT CC
STM4551_lambda_kan_F	GTT ATT TTC ATG TTG GCG CCC GAT TCC GTG TAT ATC ACC GAT AAA TAA CGA CAA TTA CCC TAA TTC CGG GGA TCC GTC GAC C
STM4551_lambda_kan_R	AAC GAT GGC AAG CGG TTT ATG GTT AAC GCT GTT GGC GTA GCA CGA TTA CGC CAA CAG GAT TGT GTA GGC TGG AGC TGC TTC
YhjH-F-XbaI	GCT CTA GAC AGG GAC CGA GGG TAA AGT T
YhjH-R-HindIII	CCC AAG CTT GTT CAG CCA GAC GAA AAA GG

1.3 Supplementary Movie Legends

Movie S1. Sal4 IgA treatment promotes agglutination of WT STm over time. WT STm 14028s was grown to mid-log phase at 37˚C and 225 rpm, harvested, washed with PBS, and standardized to an OD_{600} value of 1.0 prior to treatment with 15 µg/mL Sal4 IgA. Agglutination was filmed for 2 hours at room temperature. Tubes from left to right: untreated WT control, WT treated with 15 µg/mL Sal4 IgA.

Movie S2. Sal4-mediated agglutination occurs in a dose-dependent manner. WT STm was grown to mid-log phase at 37˚C and 225 rpm, harvested, washed with PBS, and standardized to an OD_{600} value of 1.0 prior to treatment with Sal4 IgA at the indicated concentrations. Agglutination was filmed for 1 hour at room temperature. Tubes from left to right: untreated WT control, WT treated with 3.75 µg/mL, 7.5 µg/mL, 15 µg/mL, and 30 µg/mL Sal4 IgA.

Movie S3. Sal4 IgG and IgA agglutinates WT STm over time. WT STm was grown to mid-log phase at 37˚C and 225 rpm, harvested, washed with PBS, and standardized to an OD_{600} value of 1.0 prior to treatment with 15 µg/mL Sal4 IgG or IgA. Agglutination was filmed for 2 hours at room temperature. Tubes from left to right: untreated WT control, WT + 15 µg/mL Sal4 IgG, and WT + 15 µg/mL Sal4 IgA.

Movie S4. Sal4 IgA agglutinates O5$^+$ STm over time. WT and Δ*oafA* STm strains were grown to mid-log phase at 37˚C and 225 rpm, harvested, washed with PBS, and standardized to an OD_{600} value of 1.0 prior to treatment with 15 µg/mL Sal4 IgA. Agglutination was filmed for 2 hours at room temperature. Tubes from left to right: untreated WT control, WT + 15 µg/mL Sal4, untreated Δ*oafA* control, Δ*oafA* + 15 µg/mL Sal4.

Movie S5. Sal4-mediated agglutination is dependent on flagellar-based motility. WT, Δ*motB*, Δ*flhC*, and Δ*fljB*Δ*fliC* STm strains were grown to mid-log phase at 37˚C and 225 rpm, harvested, washed with PBS, and standardized to an OD_{600} value of 1.0 prior to treatment with 15 µg/mL Sal4 IgA. Agglutination was filmed for 2 hours at room temperature. Tubes from left to right: untreated WT control, WT + 15 µg/mL Sal4, untreated Δ*motB* control, Δ*motB* + 15 µg/mL Sal4, untreated Δ*flhC* control, Δ*flhC* + 15 µg/mL Sal4, untreated Δ*fljB*Δ*fliC* control, Δ*fljB*Δ*fliC* + 15 µg/mL Sal4.

Movie S6. Sal4-mediated agglutination of 1:1 strain mixtures. WT STm (*lacZ*$^+$) was mixed 1:1 with WT (*lacZ*$^-$), Δ*oafA*, or Δ*fljB*Δ*fliC* prior to treatment with 15 µg/mL Sal4 IgA. Agglutination was filmed for 2 hours at room temperature. Tubes from left to right: untreated WT:WT control, WT:WT + 15 µg/mL Sal4, untreated WT:Δ*oafA* control, WT:Δ*oafA* + 15 µg/mL Sal4, untreated WT:Δ*fljB*Δ*fliC* control, WT:Δ*fljB*Δ*fliC* + 15 µg/mL Sal4.

Movie S7. Sal4 IgA agglutinates WT and single DGC KO strains over time. WT, Δ*STM1987*, Δ*STM2672*, and Δ*STM4551* strains were grown to mid-log phase at 37˚C and 225 rpm, harvested, washed with PBS, and standardized to an OD_{600} value of 1.0 prior to treatment with 15 µg/mL Sal4 IgA. Agglutination was filmed for 2 hours at room temperature. Tubes from left to right: untreated WT control, WT + 15 µg/mL Sal4, untreated Δ*STM1987* control, Δ*STM1987* + 15 µg/mL Sal4, untreated Δ*STM2672* control, Δ*STM2672* + 15 µg/mL Sal4, untreated Δ*STM4551* control, Δ*STM4551* + 15 µg/mL Sal4.

Movie S8. Sal4 IgA treatment agglutinates WT strains carrying plasmids overexpressing c-di-GMP modifying enzymes over time. WT + pBAD24-EV, WT + pYeaJ, and WT + pYhjH were grown to mid-log phase in the presence of 0.4% arabinose at 37˚C and 225 rpm, harvested, washed with PBS, and standardized to an OD_{600} value of 1.0 prior to treatment with 15 µg/mL Sal4 IgA. Agglutination was filmed for 2 hours at room temperature. Tubes from left to right: untreated WT + pBAD24-EV control, WT + pBAD24-EV + 15 µg/mL Sal4, untreated WT + pYeaJ control, WT + pYeaJ + 15 µg/mL Sal4, untreated WT + pYhjH control, WT + pYhjH + 15 µg/mL Sal4.
